# Next-Generation Bioinks in 3D Bioprinting: Advances, Challenges, and Emerging Opportunities

**DOI:** 10.1021/acsomega.6c07537

**Published:** 2026-08-19

**Authors:** Shruti R. Balkawade, Devika Sajeev, Usha Y. Nayak

**Affiliations:** Department of Pharmaceutics, Manipal College of Pharmaceutical Sciences, 76793 Manipal Academy of Higher Education, Manipal, India

## Abstract

3D bioprinting is an innovative technology that has advanced the field of tissue engineering and regenerative medicine by allowing the layer-by-layer deposition of cells, biomaterials, and bioactive molecules to create sophisticated biological constructs. In this context, bioinks serve as essential vehicles for creating a microenvironment that can support cell attachment, proliferation, differentiation, and maturation into tissues and organs. The current review offers a complete review of the key aspects associated with the development of 3D bioprinting bioinks, including design concepts, classification, properties, and latest trends. Several important characteristics and features of bioinks, namely, rheology, cross-linking mechanism, cell-matrix interactions, degradation process, and biofunctionalization approach, are analyzed in detail. Natural, synthetic, and hybrid bioinks with regard to printability, biocompatibility, mechanical strength, and potential for tissue regeneration are comparatively discussed. Major technologies used for bioprinting, such as inkjet printing, extrusion printing, and laser-assisted printing, are presented. Moreover, recent advances in multimaterial printing, coaxial printing, vascularization, and maturation processes in 3D bioprinting are considered. Next-generation bioinks such as nanocomposite bioinks, decellularized extracellular matrix-based bioinks, stimuli-responsive smart bioinks, 4D bioprinting materials, and artificially designed bioinks are discussed. Applications of bioprinting in skin, bone, cartilage, neural, cardiac, and tumor tissue engineering, drug screening, and personalized medicine are emphasized.

## Graphical abstract

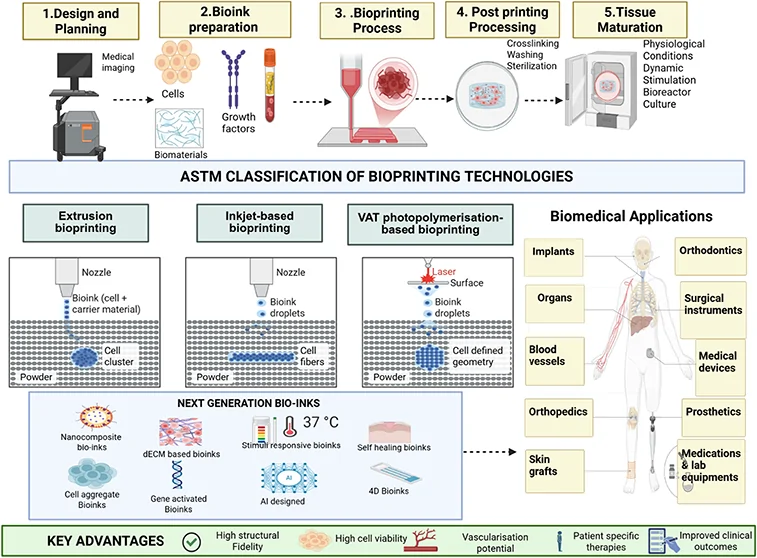

## 1 Introduction

Three-dimensional (3D) bioprinting is considered to be one of the most promising technologies in the field of tissue engineering and regenerative medicine. This is the most commonly used form of additive manufacturing technology which facilitates the creation of biological tissue such as structures with the help of layer-by-layer deposition of biomaterials, living cells, and bioactive agents into a predefined spatial order.^1–3^ According to the ASTM classification, the principal categories of bioprinting include extrusion-, jetting-, and vat photopolymerization-based bioprinting. Laser-assisted bioprinting is also widely employed as a specialized high-resolution bioprinting technique for specific biomedical applications.^4,5^ In contrast to other scaffold manufacturing methods, 3D bioprinting offers precise control over the pore geometry, cell positioning, and structure, therefore, it is highly effective and suitable for tissue fabrication.^6–8^ Bioprinting makes it possible to precisely deposit cells and materials, thereby enhancing the reproducibility of scaffolds, functionality of tissue engineering constructs, and biological incorporation. This innovative technology plays a vital role in various tissue engineering, regenerative medicine, drug screening, neural regeneration, bone tissue fabrication, vascularized tissue engineering, and organ reconstruction applications.^9,10^ In addition to tissue engineering and regenerative medicine, 3D bioprinting has become a revolutionary technique for modeling diseases, organoid production, drug discovery, toxicity testing, and personalized medicine. The bioprinted tissue structures replicate the physiological environment of the native tissue well, thereby enabling the understanding of disease processes and drug action and making minimal use of traditional animal models possible. Moreover, these engineered tissues have demonstrated significant potential in tissue engineering and regenerative medicine by enabling the fabrication of patient-specific tissue constructs for the treatment of localized injuries, chronic diseases, and organ failure. These advances highlight the expanding role of 3D bioprinting beyond tissue fabrication toward clinically relevant applications in precision medicine and regenerative therapies.^11,12^

Bioinks are printable formulations that consist of living cells, hydrogels, polymers, biomolecules, growth factors, and extracellular matrix-like material utilized in bioprinting. The main role of bioinks is to create an environment for cell attachment, cell proliferation, cell differentiation, cell migration, and tissue formation after printing.^13,14^ Ideally, bioinks must have good properties such as printability, rheology, shear-thinning properties, biocompatibility, biodegradability, mechanical integrity, and high cell viability after printing.^15,16^ Bioinks can be natural materials, which are similar to original tissues like alginate, gelatin, collagen, chitosan, fibrin, hyaluronic acid, and decellularized extracellular matrix. Synthetic polymers and nanocomposites are also included, which add electrical conductivity, mechanical integrity, and functional properties to scaffolds. Ongoing bioinks include platelet concentrate, conductive graphene, growth factors, stem cells, and bioactive nanoparticles.^17^

The development of bioinks has seen great advances, starting from basic hydrogel materials until now, where there are functional biomimicking materials capable of facilitating tissue regeneration. Bioinks initially used single-component hydrogel systems that offered minimal mechanical stability and inferior printability. Next in the development process came hybrid biomaterials made up of both natural and synthetic polymers, which are used to provide better flow behavior, biodegradation properties, structural integrity, and cell–cell interactions.^18^ Later on, gelatin methacrylate, combinations of alginate and gelatin, collagen-based products, and chitosan hybrid systems were provided for photo cross-linkage and superior printability. More advanced bioinks used nanoparticles, graphene, magnetic materials, stem cells, platelet-rich plasma, and conductive materials to give electrical conductivity, biological activity, and greater mechanical strength.^19^ Finally, more recent bioinks include smart bioinks and 4D printing materials that can respond to the different external stimuli, including pH, thermal changes, and the use of magnetic fields.

With the increasing cases of chronic disease and tissue damage, there is a need for new innovations in the regenerative science field. With the increased cases of chronic disease, injuries, organ failure, and tissue degradation, there has been an immense demand for advanced regenerative medicine techniques. Current transplantation techniques have their limitations because of donor scarcity, risk of immune rejection, expensive, time-consuming, and costly treatment processes; thus, there is a need for more advanced treatment techniques. Bone injury due to conditions such as osteoporosis, osteoarthritis, trauma, and bone tumors necessitates the use of bioactive and stable scaffolds that can promote bone formation and angiogenesis. In the case of nerve injury, neurogenic scaffolds are needed for successful repair and restoration. Additionally, there is a need for vascularized tissue within regenerative medicine to ensure viability of cells and transport of oxygen and nutrients in case of transplantation in the future. Individualized medicine is also one of the other recent trends in the field of modern medicine. The latter implies development of personalized implants, medications for children, different delivery systems, and personalized tissues using 3D printers.^20^ Drug development, disease modeling, and artificial organs represent some other applications of the technology.^21^

Even though substantial progress in tissue engineering has been made in recent years, traditional scaffold fabrication techniques still face many challenges. There are several limitations inherent in traditional scaffold fabrication techniques, limiting their applicability and effectiveness. One of these limitations is the inability to provide sufficient control over pore architecture, shape of the scaffold, and distribution of cells throughout the construct. Moreover, scaffold systems lack the necessary mechanical strength, stiffness, flexibility, and adequate control over the degradation rate that make them difficult to use surgically and reduce their efficacy. Hydrogels and conventional biomaterials also lack stability, proper vascularization, shape integrity, and cell viability after being implanted. Further issues involved with the extrusion-based process also include shear-stress injury to the cells, clogging of the nozzle, poor filament generation, and instability of the extrusion process. In addition, conventional tissue engineering techniques are unable to accurately mimic the native extracellular matrix, multicellularity, anisotropy, and dynamic mechanical environment that are essential for proper tissue development and function. Many of the currently available bioinks also have limitations related to extensive testing and limited multifunctionality.

The present review will provide a complete overview of the recent developments in the areas of 3D bioprinting technology, bioink formulation processes, scaffold manufacturing approaches, and regenerative medicine. The evolution of bioink from traditional hydrogels to advanced functional bioinks incorporating stem cells, nanoparticles, conductivity, platelet concentrates, and intelligent biomaterials is examined. Moreover, bioink fabrication using the same methods as neural scaffold fabrication using graphene, ionic liquid addition to collagen-based bioinks, magnetic bioinks, and metabolically activated polyphosphates will also be addressed. In addition, some emerging research areas such as vascular tissue engineering, bioprinted seafood, neural repair and regeneration, personalized drugs, and manipulation of cellular microenvironment mechanics will also be covered. Lastly, some potential problems concerning bioink fabrication such as issues pertaining to printability, biocompatibility, integrity, biodegradability, and clinical applications will also be analyzed. Selection of topic is relevant regarding transforming the nature of 3D bioprinting technology since it not only represents a sophisticated tool of tissue engineering but also addresses the challenges related with traditional techniques of regenerative medicine.

## 2 Design Principles of Bioinks

### 2.1 Rheological Requirements

The key design factor for obtaining efficient printability is the rheological properties of bioinks. For stable printing that maintains shape, a bioink should have properties such as shear thinning, meaning that the fluid shows decreased viscosity under applied shear stress while it passes through the printer’s nozzle but rapidly regains its viscosity after deposition.^22^ While viscoelasticity regulates the filament’s ability to retain its structure after deposition, dynamic viscosity influences the flow characteristics inside the printing nozzle.^23^ For fluids that are insufficiently thin or excessively viscous to achieve sufficient structure preservation, the latter problem is especially troublesome.^24^

Shear-thinning behavior is perhaps one of the most critical rheological properties since it enables bioinks to pass easily through the nozzle due to shear stresses, but then regain their viscosity when deposited.^25^ The printability of tubular constructs relies greatly on bioink viscosity, extrusion flow rate, temperature during printing, and composition of the supporting bath.^26^ Physical cross-linked gelatin bioinks supplemented with gellan gum and Laponite demonstrate better rheological and shape stability properties without needing any chemical cross-linkers.^27^ Starch-gelatin bioink nanoparticles are highly thixotropic and shear-thinning bioinks ideal for extrusion printing.^28^ Thixotropy and shear recovery are of equal importance in the field of rheology. The bioink needs fast recovery when shear stress is applied during extrusion in order to preserve the architecture of the scaffold and prevent the spreading of filaments. In the experiments that are conducted on bioinks based on gelatin-alginate and those containing graphene, it was seen that thixotropic properties play a vital role in the quality of the scaffolds that are produced using bioprinting technology. Rheological optimization is totally dependent upon biological functionality since high viscosity helps achieve better structural stability but also can hinder cell movement and growth. The extent to which tendon is digested with decellularized ECM makes a significant difference, it impacts the viscosity, extrusion, and printability. Reduced digestion helps to maintain engine control module (ECM) structural integrity and increases the printing efficiency.^29^ Thermal treating and saponification of hydrogel bioinks sometimes result in more stable rheology, improving printability in thermal inkjet bioprinting.^30,31^

### 2.2 Cross-Linking Mechanisms

Cross-linking is an important factor that enables the transformation of liquid bioink into a stable 3D construct. The selection of the cross-linking technique affects properties such as mechanical strength, degradability, printability, and cell viability. Ionic cross-linking is a technique in the extrusion process because it offers fast formation under mild conditions. For example, materials like alginate and gelatin can be used with calcium ions. On the other hand, ionic cross-linking leads to variations in mechanical strength owing to ion diffusion.^32^ Cross-linking through chemical cross-links includes the development of covalent links between polymer chains using radical polymerization, condensation, irradiation, or chemicals for cross-linking. Such techniques normally result in high mechanical strength, but they can become cytotoxic when the wrong cross-linking occurs.^24^ Photocross-linking has received much attention due to the ability of light-sensitive materials such as GelMA, hyaluronic acid methacryloyl (HAMA), and silk fibroin methacryloyl (SFMA) to develop a network of hydrogels when exposed to UV or visible light along with the photoinitiators. Dual-cross-linking systems that utilize both photo- and ionic cross-linking systems have been proposed.^33^ Photocross-linking enables rapid stabilization of bioinks and improves structural fidelity during printing.^34,35^ Direct in situ photo-cross-linking during extrusion reduces filament collapse and enables fabrication of free-standing structures.^36^ Physical cross-linking provides a safer alternative to chemical cross-linkers and reduces the risk of cytotoxicity.

### 2.3 Cell–Material Interactions

The effectiveness of the biological behavior of the bioinks depends mainly on the nature of the interaction between cells and material. Cell–material interaction involves mimicking the original conditions by ensuring that the matrix is able to transmit biochemical signals involved in cell adhesion, proliferation, migration, differentiation, and tissue maturation processes.^37^ Natural biomaterials including collagen, fibrin, hyaluronic acid, and decellularized extracellular matrix offer an added advantage of offering natural materials for cellular interaction. Decellularized extracellular matrix-based bioinks have been widely used due to their capacity to maintain native biochemicals without any significant triggering of immunological reactions. The bioinks promote cellular interaction that leads to cellular adhesion, proliferation, and differentiation. Mechanical and biochemical prompts generated by bioinks play a major role in determining the function of the bioinks in the promotion of cell behavior and functionality. ECM-mimicking (extracellular matrix) materials have also been known to regulate stem cell fate. Bioinks should provide biochemical and mechanical aspects that regulate cell adhesion, migration, proliferation, and differentiation.^38^ Recombinant protein and ECM-inspired bioinks improve cell compatibility and support long-term cell survival. Silk fibroin-based bioinks promote excellent cell attachment because of their natural protein structure. The bioactive scaffold structures with the periodontal cells improve cell attachment, proliferation, and regeneration capacity.^39^ The functional bioinks should create a proper microenvironment to support cell viability, attachment, proliferation, and differentiation postprinting process.^40^ Physical stimuli produced by bioinks govern cell shape, migration, and differentiation.^4^ Cell density plays an important role in determining bioink effectiveness, as high cell density causes alterations in rheological behavior, requiring optimization of both bioink materials and also printing techniques.

### 2.4 Degradation Kinetics

Controlled degradation becomes a delicate aspect of tissue-engineered scaffolds, since the speed of degradation needs to match the speed of tissue formation. Otherwise, the structure can either degrade before the process of tissue formation is over, or vice versa, it can remain intact after the tissue is fully formed. Depending on the type of material, its degradation rate might be affected. For example, hydrogels, including hyaluronic acid, degrade quickly and usually require modification to enhance their stability. Gelatin bioink scaffolds typically demonstrate rapid degradation and cannot sustain their structures for a long time. Synthetic materials, such as PLA, PCL, and PEG, degrade slowly and provide sustained mechanical support, however, their bifunctionality might need enhancement.^33^ Degradation is affected by the type of material used, the degree of cross-linking, the environment, and enzyme action. Hence, depending on the tissue targeted, the degradation should be adjusted.

### 2.5 Biofunctionalization Strategies

One of the approaches that have been utilized extensively to enhance biological properties is biofunctionalization. With respect to growth factors, biomolecules, peptides, nanoparticles, and drugs in the bioink formulation lead to increased biological activity and regeneration capacity.^41^ Nanocomposites with nanoparticles can improve mechanical strength, printability, and bioactivity of the bioinks as well as act as drug-delivery devices. Growth factors incorporated in the bioink formulations direct stem cell differentiation and lead to faster tissue-specific regeneration. On the other hand, functionalizing the bioink formulation with peptides, such as the incorporation of arginylglycylaspartic acid (RGD) peptides in polyethylene glycol (PEG) bioinks increases cell adhesion and proliferation.^42^ Graphene oxide incorporation enhances both biological activity and mechanical performance in vascular bioinks.^43^

Further advanced strategies for biofunctionalization involve the inclusion of extracellular matrix components, revascularized spheroids, microspheres with cells inside, and gradients of cells to replicate natural tissue complexity. Moreover, smart bioinks incorporating self-healing hydrogels, nanomaterials, and stimuli-responsive polymers have been developed which are capable of providing dynamic biological activity without compromising the printability and stability of the bioinks. As a result, the process of biofunctionalization makes the bioinks active biological agents capable of modulating cell behavior and leading to better regeneration.

### 2.6 The Bioprinting Technologies

#### 2.6.1 Extrusion-Based Bioprinting

Extrusion-based bioprinting is one of the most commonly used bioprinting technologies due to its ability to construct complex 3D tissue structures with the use of diverse natural and synthetic bioinks. In this process, cell-laden bioinks are extruded in the form of continuous filaments by means of pneumatic, piston, and screw-driven dispensers, which allows printing of high-viscosity materials and cell concentrations.^1,17,44^ In spite of all these merits, the extrusion-based bioprinting method imposes a considerable mechanical stress on encapsulated cells while printing. Of all the available methods for bioprinting, extrusion bioprinting is the one that imposes the largest amount of shear stress on the cells, because they are being forced out of a narrow nozzle under pressure and spend much time inside. High mechanical stress causes disruption of the cell membrane, changes in the cytoskeleton, and apoptosis.^45^ The level of shear stress depends mainly on extrusion pressure, nozzle diameter, nozzle shape, printing speed, and bioink viscosity. The higher the extrusion pressure, the better the filament structure and print quality will be, although at the same time more shear stress will be imposed on cells. In addition, a smaller nozzle diameter provides higher precision of printing but also larger shear stresses.^46,47^ Bioink rheology plays an important role in providing cellular protection during extrusion. The shear-thinning properties of hydrogel enable lowering viscosity under pressure, thus ensuring a less stressful extrusion process. After deposition, these materials quickly recover their viscosity, preserving structural integrity and providing a suitable microenvironment for cell adhesion and proliferation.^48^ Among other hydrogels, GelMA, alginate, collagen, hyaluronic acid, and decellularized extracellular matrix prove themselves to be highly printable and cytocompatible due to their good rheological characteristics. At the same time, highly viscous bioinks are more difficult to extrude, while too low viscosity causes problems with shape fidelity and construct stability. Therefore, tuning bioink viscosity becomes crucial in obtaining a balance between printability and cell survival.^49^

Printing temperature also impacts cell viability, especially in case of thermosensitive hydrogels. Low temperatures cause less thermal stress; however, extended residence of cells in the cartridge leads to decreasing metabolic activity due to the depletion of nutrients and mechanical load on cells.^50^ Cell density is yet another key process parameter. While high cell densities facilitate cell–cell interactions and ECM deposition, they also lead to increased viscosity and resistance to extrusion, which raises shear stress and the risk of nozzle blockage.^51^ The subsequent step of the process is cross-linking of the construct. Overly strong cross-linking makes the matrix stiff and impedes the diffusion of nutrients, while weak cross-linking causes the collapse of the structure. Optimal cross-linking ensures the maintenance of the hydrogel structure, keeping a high cell viability and promoting tissue maturation. The latest developments include the design of low-shear nozzles, sacrificial baths, shear-protected bioinks, and computer simulations of the printing process. They allow reducing mechanical damage at the stage of extrusion and result in high cell viabilities (exceeding 85–90%) after printing of various tissues.^17,46^

#### 2.6.2 Jetting-Based Bioprinting

**Jetting-based bioprinting** is a noncontact printing process involving the generation and deposition of picoliter- to nanoliter-sized droplets of living cells suspended within bioinks on a designated substrate. In comparison to the extrusion bioprinting approach, this method exposes cells to reduced mechanical forces and yields a greater cell viability after printing. Nevertheless, its use is limited to low-viscosity bioinks.^52,53^ Inkjet bioprinting uses thermal, piezoelectric, and electrostatic actuation principles to produce the bioink droplets. Despite being subject to localized thermal stress during the printing process, thermal inkjet printers heat bioinks for only a few microseconds, which does not cause significant damage to cell integrity under optimal printing conditions. Piezoelectric inkjet printers do not cause thermal exposure; however, pressure waves produced in these devices may harm cell membrane structure in the case of too high driving voltage and/or frequency.^13^ The droplet ejection velocity is among the most important factors affecting cell viability. With greater ejection velocities, printing speed and droplet positioning accuracy are increased, but mechanical forces become stronger at the time of droplet formation and deposition on the substrate, which can cause a rupture of membranes and decrease in cell proliferation. Thus, optimization of actuation voltage, pulse duration, and firing rate becomes vital in order to reduce mechanical forces. In addition to droplet ejection velocity, the droplet volume rapidly influences postprinting performance. Smaller droplets provide higher spatial resolution but may expose encapsulated cells to higher shear and extensional stresses during droplet formation, thereby increasing the risk of membrane damage. Conversely, larger droplets offer improved cellular protection by reducing mechanical stress but may compromise the printing resolution and positional accuracy. Moreover, the combined effects of droplet velocity, droplet volume, bioink viscosity, surface tension, nozzle diameter, and actuation parameters determine important hydrodynamic parameters such as the Weber number (We) and Reynolds number (Re), which govern droplet formation, breakup, and impact behavior, as well as cell survival. Therefore, careful optimization of droplet characteristics and fluid properties is essential to achieve stable droplet generation, high printing fidelity, and maximum postprinting cell viability.^54^ Rheological characteristics of the bioink significantly affect droplet formation and printing processes.^53^

Low-viscosity bioinks with an appropriate surface tension form stable and uniform droplets, while high-viscosity bioinks can lead to nozzle clogging and irregular droplet formation. Therefore, almost all printable bioinks have viscosities lower than about 10–15 mPa·s. Another factor that affects cellular behavior in the course of printing is the nozzle diameter. A smaller nozzle diameter helps achieve greater print resolution; however, the cells experience more localized shear forces and face a higher risk of clogging in case of high cell concentrations or large cell aggregates. Selecting the appropriate nozzle diameter based on cell type and bioink is necessary for precise deposition and cell survival. A high cell concentration plays a significant role in printability and biological performance of bioinks. The higher concentration of cells leads to enhanced cell–cell communication and tissue formation; however, very concentrated bioinks cause droplet instability, sedimentation, and nozzle clogging, which lead to uneven cell distribution.^50^ The latest improvements in inkjet bioprinting include optimization of waveforms, multiple-nozzle bioprinting, and bioinks with advanced rheological properties, allowing for highly reproducible printing of various cell types while preserving cell viability of over 90%. Inkjet bioprinting appears to be especially promising in skin, vascular, and high-throughput tissue engineering.^13^

#### 2.6.3 Vat Photopolymerization-Based Bioprinting

Vat photopolymerization includes stereolithography (SLA), digital light processing (DLP) and continuous liquid interface production (CLIP), where photosensitive bioinks undergo selective polymerization via controlled light exposure. This technique offers high-resolution printing capabilities, a smooth surface structure, and rapid formation of complicated 3D structures. The printed scaffold is made without extruding the material mechanically through a nozzle; thus, the cells are subjected to low shear stress, which increases cell viability compared to extrusion-based methods.^18,55,56^ Although cells are subjected to low shear stress, the light exposure itself poses some risks of phototoxicity, which should be considered. Too much ultraviolet radiation or too much time of light exposure leads to the formation of reactive oxygen species (ROS), which destroy DNA, proteins and cell membranes and provoke apoptosis. It is important to optimize the light wavelength, intensity, and exposure time to keep the cells alive. The choice of photoinitiator is yet another vital parameter that determines cytocompatibility. While conventional photoinitiators used for UV curing are efficient initiators of polymerization, they may generate high amounts of free radicals that hinder cell metabolism. There are several biocompatible photoonitiators that have been developed to improve cytocompatibility while maintaining efficient photo-cross-linking. Lithium phenyl-2,4,6-trimethylbenzoylphosphinate (LAP) is one of the most widely used photoinitiators because of its high water solubility, rapid polymerization kinetics, and ability to be activated by visible or near-UV light, thereby reducing phototoxicity. Irgacure 2959 is another commonly used photoinitiator that exhibits relatively low cytotoxicity at appropriate concentrations and has been extensively employed in hydrogel-based bioprinting. Visible-light photoinitiating systems, such as Eosin Y in combination with triethanolamine and *N*-vinylcaprolactam, as well as tris­(2,2′-bipyridyl)­dichlororuthenium­(II)/sodium persulfate (Ru/SPS), further enhance cell viability by minimizing UV exposure while providing efficient cross-linking. More recently, diphenyl­(2,4,6-trimethylbenzoyl)­phosphine oxide (TPO-L) has also attracted attention because of its high curing efficiency and improved cytocompatibility. The selection of an appropriate photoinitiator should therefore consider its absorption wavelength, free-radical generation efficiency, concentration, and overall compatibility with encapsulated cells.^57^

In contrast, low-toxicity photoinitiators that respond to visible light, like lithium phenyl-2,4,6-trimethylbenzoylphosphinate (LAP), show greater cytocompatibility along with quick efficiency of the curing process.^58,59^ Moreover, the concentration of these photoinitiators should be carefully optimized to balance polymerization efficiency, curing depth, and cytocompatibility. Low concentrations lead to incomplete polymerization and unstable structure, while high concentrations lead to higher free radical generation and toxicity. Some studies have proved that using lower concentrations of photoinitiators and optimizing light curing parameters improve proliferation and maturation of tissues.^60^ However, excessively high photoinitiator concentrations may reduce light penetration due to increased light absorption and scattering, thereby limiting the effective curing depth while simultaneously generating excessive free radicals that can impair cell viability. Conversely, insufficient photoinitiator concentrations lead to incomplete polymerization, a shallow curing depth, poor mechanical stability, and reduced structural integrity. Therefore, an optimal photoinitiator concentration should be selected to achieve an appropriate balance among curing efficiency, structural stability, and cytocompatibility.^18,61–63^

## 3 Types of Bioinks

Bioinks are the basic building blocks of 3D bioprinting technology that is responsible for encapsulating living cells, biomolecules, and other bioactive molecules in order to construct tissue-engineered structures. The types of bioinks can be divided based upon the nature of the materials present in it, and there are mainly three broad classes of bioinks, such as natural bioinks, synthetic bioinks, and hybrid bioinks (Table 1). Figure 1 presents the distinction between bioinks and biomaterial inks and the overall 3D bioprinting workflow from ink preparation to tissue regeneration. Every bioink category has certain benefits and drawbacks concerning its ability to print and biological properties. The rheological characteristics of bioinks are the most important design criteria for achieving effective printability and proper functioning. Ideally, a bioink must be shear-thinning in nature, where as the fluid exhibits reduced viscosity under applied shear stress when passing through the printer’s nozzle, it quickly restores its viscosity postdeposition for stable printing with preserved shape. Dynamic viscosity affects flow characteristics within the printing nozzle, while viscoelasticity controls the capacity of the filament to maintain its shape after being deposited. The latter issue is particularly problematic for fluids that are overly viscous or too thin to achieve adequate structure preservation. This classification is primarily based on the origin and composition of the biomaterials used in bioink formulation.

**Figure 1:**
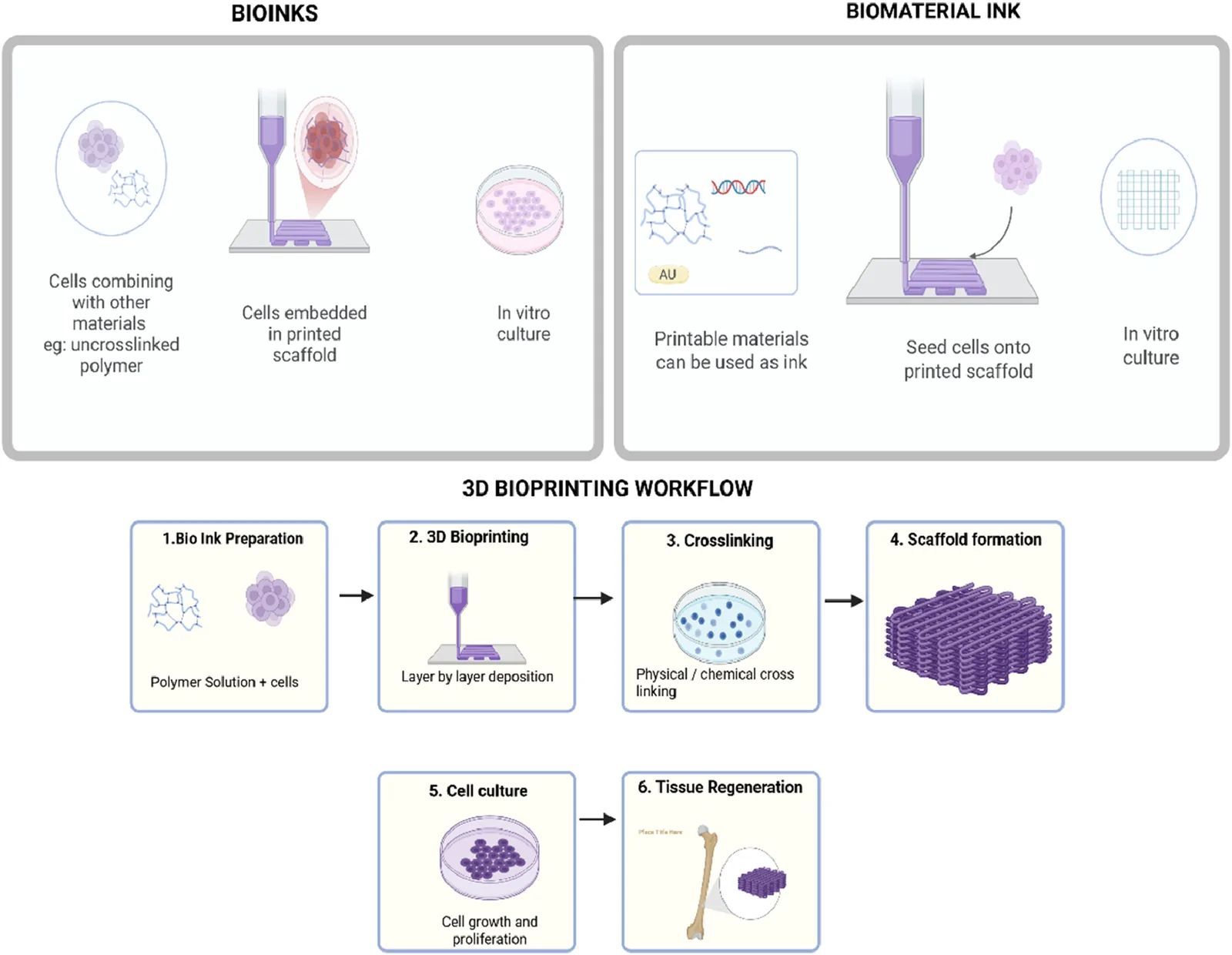
Bioink and biomaterial with 3D Bioprinting workflow (Figure created using BioRender).

**Table 1:** Types of Bioinks Used in 3D Printing

**Type of bioinks**	**Representative material**	**Major components**	**Properties**	**Advantage**	**Disadvantage**	**Major applications**
**Natural bioinks**	Alginate	It is a natural polysaccharide from algae	Ionic cross-linking, shear thinning, biocompatible	Very good printability, cost-effective and easy gelation	Poor cell adhesion and weak mechanical strength	Cartilage, vascular soft tissue engineering^72^
	Collagen	ECM protein	Biocompatibility and cell recognition motifs	Excellent cell attachment and proliferation	Low viscosity and poor mechanical stability	Skin, cardiac, neural tissue engineering
	Gelatin	Denatured collagen	Thermoresponsive and biodegradable	Biocompatible and cost-effective	Weak structure and rapid degradation	Skin, cartilage, and wound healing^73^
	GelMA	Methacrylated gelatin	Photocross-linkable and tunable mechanics	Excellent printability and cell viability	Requires photoinitiators and UV exposures	Vascular, cardiac, cartilage engineering
	Hyaluronic acid	ECM glycosaminoglycan	Hydrophilic, bioactive	Supports cell migration and proliferation	Rapid degradation and weak mechanical properties	Neural and cartilage regeneration^74^
	Fibrin	Blood clotting protein	Bioactive and cell interactive	Stimulates the angiogenesis and wound healing	Faster degradation	Vascular and skin regeneration
	Chitosan	Marine polysacchride	Antibacterial and biodegradable	Biocompatible and bioactive	Poor printability	Bone and cartilage
	Silk fibrion	Natural protein polymer	Tunable degradation and strong mechanics	Excellent mechanical strength and biocompatibility	sSlow gelation	Bone cartilage and vascular tissue engineering^75^
	dECM	Decellularized extracellular matrix	Tissue-specific biochemical cues	Mimics native ECM	Batch to batch variability and poor mechanical strength	Organ-specific tissue engineering
**Synthetic bioinks**	PEG	Synthetic polyether	Tunable properties, nonimmunogenic	Highly reproducible and controllable	Poor cell adhesion	Drug delivery, tissue engineering^76^
	PEGDA	Photocross-linkable PEG derivative	Tunable stiffness and degradation	Excellent structural fidelity	Low bioactivity	Vascular and cardiac constructs
	PCL	Polyester polymer	Slow degradation, high strength	Load-bearing applications	Poor cell interaction	Bone and cartilage engineering^77^
	PLA	Biodegradable polyester	Mechanical support	Easy fabrication and stability	Hydrophobic surface	Orthopedic scaffolds
	PLGA	Copolymer of PLA and PGA	Controlled degradation	FDA-approved biomaterial	Limited bioactivity	Drug delivery and regenerative medicine
	PVA	Synthetic polymer	Hydrophilic and flexible	Good mechanical properties	Requires biofunctionalization	Cartilage and soft tissues^78^
	PNIPAM	Thermoresponsive polymer	Temperature-sensitive behavior	Smart bioink applications	Limited cell interaction	Stimuli-responsive systems
**Hybrid bioinks**	Alginate + gelatin	Natural-natural hybrid	Improved rheology and printability	Better structural stability and cell support	Requires optimization	Skin, cartilage, vascular tissues^79^
	Collagen + PEG	Natural-synthetic hybrid	Enhanced bioactivity and strength	Improved mechanical integrity	Complex formulation	Tissue engineering
	GelMA + SFMA	Dual-cross-linking hybrid	Excellent printability and stability	High cell viability and structural fidelity	Expensive preparation	Advanced tissue constructs
	Chitosan + PCL	Natural-synthetic hybrid	Enhanced mechanics and bioactivity	Suitable for hard tissues	Processing complexity	Bone regeneration
	Hyaluronic acid + PEG	Hybrid hydrogel	Tunable degradation and mechanics	Improved tissue mimicry	Multiple processing steps	Cartilage and neural tissues^80^
**Nanocomposite bioinks**	Graphene oxide bioinks	Graphene nanosheets	Conductive, mechanically reinforcing	Enhanced cell signaling and conductivity	Potential nanotoxicity concerns	Neural, cardiac and vascular tissues
	Carbon nanotube bioinks	CNTs	Electrical conductivity	Promotes neural and cardiac function	Safety concerns	Neural and cardiac regeneration
	Hydroxyapatite nanocomposites	Bioactive ceramic nanoparticles	Osteoconductive	Bone mineral mimicry	Brittle at high loading	Bone tissue engineering
	Nanoclay bioinks	Laponite and nanoclay	Rheological enhancement	Improved printability and strength	Optimization required	Bone and cartilage applications^81^
**Functional bioinks**	Stem cell-laden bioinks	MSCs, iPSCs, stem cells	Regenerative potential	Tissue-specific differentiation	Regulatory challenges	Personalized regenerative medicine
	PRP bioinks	Platelet-rich plasma	Growth-factor rich	Accelerated healing and angiogenesis	Batch variability	Wound healing and bone repair
	Growth factor bioinks	VEGF, BMP, TGF-β	Biological signaling	Enhanced tissue regeneration	High cost and instability	Vascularization and tissue repair
**Smart bioinks**	Thermoresponsive bioinks	Temperature-sensitive polymers	Sol–gel transition	Easy handling and printing	Temperature dependence	Drug delivery and tissue engineering
	pH-responsive bioinks	pH-sensitive hydrogels	Controlled swelling and degradation	Targeted therapeutic delivery	Complex formulation	Drug-delivery systems
	Photoresponsive bioinks	Light-sensitive polymers	Controlled cross-linking	High spatial precision	Light exposure limitations	4D bioprinting^82^
	Magnetic bioinks	Magnetic nanoparticles	External control capability	Remote stimulation and positioning	Nanoparticle toxicity concerns	Targeted tissue engineering
**Next-generation bioinks**	AI-designed bioinks	Machine learning optimized formulations	Predictive performance modeling	Faster optimization and reduced experimentation	Early stage technology	Precision bioprinting
	4D Bioinks	Stimuli-responsive smart materials	Shape transformation over time	Dynamic tissue adaptation	Manufacturing complexity	Adaptive implants and regenerative medicine
	Multifunctional bioinks	Combined nanomaterials, cells and growth factors	Simultaneous biological and mechanical functionality	Mimics native tissue complexity	High production cost	Advanced organ engineering^83^

### 3.1 Natural Bioinks

Natural bioinks are produced from naturally available biomaterials that mimic the composition of original ECM. They provide exceptional biological materials for cell adhesion, growth, migration, and differentiation. Alginate has emerged as one of the most common natural bioinks because of the simple cross-linking process involving Ca2+ ions and good printability of the material. However, this biomaterial is unable to support cell growth and usually must be blended with other biomaterials. Collagen is considered another essential natural bioink because collagen is a primary structural protein of native ECM. Collagen offers excellent recognition sites for cells but lacks adequate mechanical strength and thus needs to be reinforced with cross-linking agents. Gelatin and GelMA are mainly used because of their good biocompatibility, ability to support cell adhesion, and photo-cross-linking property. Bioinks based on GelMA have been successfully applied to fabricate soft tissues and blood vessels. Fibrin, hyaluronic acid, decellularized extracellular matrix (dECM), chitosan, silk fibroin, and agarose are among other natural bioinks. They exhibit remarkable biological functionality but lack proper mechanical strength, and they degrade quickly. Besides, printed shapes using these biomaterials may not retain their original shape after printing. Gelatin remains one of the most widely used natural bioinks because it supports cell attachment and mimics ECM properties. Silk fibroin exhibits excellent biocompatibility, tunable degradation, and adjustable mechanical properties, making it an attractive natural bioink. The decellularized extracellular matrix (dECM) bioinks replicate the natural composition of the tissues and supply the tissue-specific biochemical cues.^64,65^ Bioinks made of silk fibroin demonstrate outstanding biocompatibility, mechanical strength, and regulated biodegradability. ECM-derived bioinks provide tissue-specific biological signals and mimic the native extracellular matrix environment.^66^

### 3.2 Synthetic Bioinks

The major advantage of artificial bioink is that it is based on artificially synthesized polymers, which offer better mechanical performance, reproducibility, and controllability when compared with natural biomaterials. The material’s chemical structure may be accurately adjusted in order to attain optimal rheology, degradation rate, and stability. Among the most studied artificial bioinks are polyethylene glycol (PEG) and polyethylene glycol diacrylate (PEGDA). PEG and PEGDA are known for their great adjustability and photochemical cross-linking properties, yet their low biological activity does not allow cell attachment. Polycaprolactone (PCL) is one of the most popular materials in bone and cartilage tissue engineering due to its high mechanical properties and slow degradation. However, PCL lacks cell attachment capability. PVP-based bioinks improve cell homogeneity and reduce cell sedimentation during printing^67^

Polylactic acid (PLA), poly­(lactic-*co*-glycolic acid) (PLGA), polyethylene glycol diacrylate (PEGDA), poly­(vinyl alcohol) (PVA), polycaprolactone (PCL), and poly­(*N*-isopropylacrylamide) (PNIPAM) also belong to the class of artificial biomaterials used in bioprinting techniques. These biomaterials are characterized by high stability, and they have to be biofunctionalized.^68^ PEG-based hydrogels provide tunable mechanical properties and controlled photo-cross-linking behavior.

### 3.3 Hybrid Bioinks

Hybrid bioinks are created through the mixing of two or more types of biomaterials in order to address problems related to individual materials. The idea is to incorporate the biological features of natural biomaterials, and at the same time, it provides the required mechanical strength and flexibility with synthetic polymers. Modern achievements show that the application of hybrid bioinks can dramatically enhance the printability, structure, degradability, and functional capacity of printed products. The combination of methacrylate silk fibroin with GelMA is an innovative technique. In this case, there is an occurrence of double cross-linking, namely, through thermal and photopolymerization processes. This provides increased stability of the formed scaffold as well as the viability of cells in it. It is possible to create complex tissues with the use of such a hybrid ink because of enhanced properties of the latter. Hybrid bioinks combine the biological advantages of natural polymers with the mechanical strength and reproducibility of synthetic polymers. Chitosan-collagen-hydroxyapatite composite bioinks demonstrate enhanced mechanical performance for dental tissue engineering.^69^ A combination of the ECM elements and the artificial polymers leads to enhanced biological and mechanical properties.^70^ Hydrogel composites allow for achieving a balance between printability and cell performance characteristics that cannot be obtained with single-component materials.^71^ Examples of hybrid bioinks involve GelMA + silk fibroin methacrylate (SFMA), alginate + gelatin, collagen + PEG, chitosan + PCL, and a combination of natural and synthetic bioinks.

The use of both natural and synthetic biomaterials in bioinks is the most attractive technology. Natural materials offer bioactivity that allows the cells to attach, multiply, and differentiate; synthetic polymers give mechanical integrity, degradation capability, and processability. One example would be collagen hydrogel functionalized by PEG, which offers enhanced mechanical stability but remains biocompatible and retains its favorable cell behavior features.^33^ Another good example is alginate-GelMA combinations; they are capable of rapid ionic gelation and photopolymerization into stable and active structures.^33^

#### 3.3.1 Manufacturing Feasibility of Hybrid Bioinks

In particular, hybrid bioinks are characterized by great manufacturing feasibility due to the possibility of optimizing the ink composition based on desired tissue needs. The use of dual-cross-linking, rheological property adjustment, and additives results in scalability, and high printability, and biological performance. Synthetic materials provide increased consistency and reproducibility, while natural biomaterials provide the biological signals necessary for regeneration. The development of extrusion printing technology, photo-cross-linking techniques, and biofunctionalization approaches has allowed for large-scale production of hybrid bioinks in recent years. Therefore, it is possible to say that hybrid bioinks are considered the most important technology for the future development of biofabrication due to their ability to combine both the biological performance of natural materials and the mechanical performance of synthetic materials in one product.

## 4 Properties of an Ideal Bioink

The success of the process of 3D bioprinting mainly depends on certain features of the bioink that is used. An ideal bioink should have biological, physical, chemical, and mechanical properties to ensure that the printing process is effective but still preserve cell viability and construct integrity.

### 4.1 Biocompatibility

Biocompatibility is one of the key characteristics for the bioink to be considered ideal. The material should not cause any adverse biological effects, including toxicity and immunogenicity, but it should create a positive environment for cells. In particular, it is vital for cells to attach, proliferate, migrate, and differentiate. Collagen, gelatin, hyaluronic acid, fibrin, and decellularized extracellular matrix are often utilized since they are similar to the original extracellular matrix and promote cell behavior. Good biocompatibility is necessary for the tissue functionality and regeneration. The characterization of the bioink biocompatibility should include the cytotoxicity, proliferation, differentiation, and immune response for assessment purposes. Cell-laden hydrogels must maintain high viability during extrusion, cross-linking, and long-term culture.^84^ Bioinks should maintain high cell viability during extrusion and after cross-linking. PVP-containing bioinks significantly improve cell survival and homogeneity during drop-on-demand printing.

### 4.2 Printability

The concept of printability describes how well the bioink can be precisely dispensed from a printer nozzle while ensuring proper geometrical and structural characteristics of the printed scaffold structure. Printability of bioinks requires controlled behavior under extrusion and appropriate shape retention after dispensation. The properties affecting printability of bioinks include viscosity, surface tension, shear-thinning properties, nozzle suitability, and cross-linking. Good printability results in the accurate construction of complex three-dimensional structures. Printability depends on the viscosity, yield stress, cross-linking rate, filament stability, and shape retention also after deposition. Resolution limitations remain a major challenge to fabricate highly complex tissues and microvascular structures.^85^ Printability depends on the viscosity, surface tension, gelation kinetics, and extrusion parameters. New gelatin-based bioinks exhibit excellent printability while maintaining structural stability under physiological conditions.

### 4.3 Mechanical Strength

The desired property of bioinks includes the ability to have enough mechanical strength to retain their structural integrity when printing. It is necessary for the mechanical properties of the bioinks to be like the mechanical properties of the tissues, which will help them be able to support cell growth. Lack of mechanical strength can cause bioinks to deform, collapse, and degrade prematurely after implantation. The use of synthetic polymers, nanomaterials, graphene, and composite biomaterials improves the mechanical properties of the scaffolds. Graphene oxide improves the viscoelasticity and mechanical strength of collagen-based vascular bioinks. Hydroxyapatite-reinforced chitosan/collagen constructs showed improved compressive strength and toughness. Cartilage and bone tissue engineering require bioinks that have enhanced compressive strength and stiffness while maintaining biological performance.^11^ The nanocomposite improves mechanical integrity without significantly compromising cell viability.

### 4.4 Biodegradability

Biodegradability is an important attribute that ensures that the bioink scaffold gradually breaks down in response to the formation of new tissue. The optimal rate of biodegradation must be balanced with the tissue regeneration processes to ensure consistent scaffolding through the entire repair period. On one hand, rapid degradation could disrupt scaffold integrity, while on the other hand, too slow of a breakdown process may impede tissue remodeling and integration. Naturally occurring biomaterials such as gelatin and hyaluronic acid tend to degrade quickly, whereas synthetic materials such as PLA, PCL, and PEG offer longer structural durability. Silk fibroin and gelatin bioinks exhibit controllable biodegradation rates suitable for tissue regeneration.^32^

### 4.5 Cell Viability

High cell viability is crucial during and after the bioprinting process to produce a viable tissue construct. A good bioink would provide protection against mechanical stress, shear forces, and environmental harm to the cells within. Additionally, the scaffold matrix must have enough nutrients and an oxygen supply to sustain cell viability and promote proliferation and metabolic activity. Researchers have found that certain hydrogel matrices and ECM-mimicking bioinks allow high postbioprinting cell viability with the formation of the tissue structure. More than 90% viability was observed in photo-cross-linked cardiac tissue bioinks under optimized printing conditions. Corneal keratocytes maintained a high viability after collagen-based bioprinting.

### 4.6 Sterility

Sterility most important characteristic in relation to all bioinks used in biomedical and clinical applications. Bioinks need to be free from any microorganisms, toxins, fungi, viruses, and other potential contaminants that might harm cells or affect the survival rate. Sterilization and aseptic techniques are critical at each stage of the process, in the production of bioinks and bioprinting procedures. Ethylene oxide sterilization preserves rheological and biological properties better than autoclaving.^86^

### 4.7 Viscosity

Viscosity is an important factor when assessing the performance of bioinks in the printing process. The best bioink formulation should be at an optimal level of viscosity that enables extrusion without compromising the integrity of the structure that was printed. Formulations with too low or too high viscosity have adverse effects on the process. It is advantageous to use shear-thinning materials with decreased viscosity in extrusion, followed by rapid recovery after extrusion.

### 4.8 Gelation Ability

The ability of the bioink to be capable of forming from liquid state to a stable three-dimensional hydrogel structure after deposition is known as gelation ability. It is important for the proper functioning of the scaffold in terms of maintaining its shape, avoiding its collapse, and providing an environment for the cells. There are various methods of gelation such as ionic cross-linking, photo-cross-linking, enzymatic cross-linking, thermal gelation, and chemical cross-linking depending upon the type of biomaterial used. The better the gelation ability of the bioink, the more stable, mechanically superior, and functional it is. In general, the perfect bioink would incorporate all the above properties along with excellent biocompatibility, printability, mechanical stability, biodegradability, cell viability, sterility, optimum viscosity, and gelation ability.^30^

## 5 3D Bioprinting Techniques and Biofabrication Strategies

### 5.1 Jetting-Based Bioprinting

Inkjet bioprinting is a droplet-based bioprinting technology that fabricates three-dimensional biological materials through the creation of controlled microdroplets of bioinks on the surface of a substrate in a layer-by-layer fashion. This is done by using various methods such as thermal, piezoelectric, electromagnetic, and valve-driven inkjet printing systems.^87,88^ This type of bioprinting technology is well-suited for use in low-viscosity bioinks and allows for fast printing along with highly ordered resolution. Smaller diameter nozzles of between 50–100 μm help in achieving precise control in depositing cells and biological materials, allowing for the creation of complex microarchitectures and vascular networks through inkjet bioprinting. Inkjet bioprinting provides high precision but requires low-viscosity bioinks.^54^

Piezoelectric inkjet printing systems use the energy produced from acoustic waves to increase precision and enhance cell viability. In thermal inkjet systems, high-frequency pulses of heat are used to eject the droplets without negatively impacting cell viability. Valve-driven systems also have enhanced cell viability since thermal stress during the process is minimized; thermal inkjet bioprinting offers rapid fabrication and high resolution but requires low-viscosity formulations.^61^ High droplet speed might decrease viability due to mechanical stress during impact. The importance of inkjet bioprinting in modern biotechnology cannot be overstated as it has found wide application in tissue engineering, drug screening, and high-throughput biological studies. However, limitations in this technology include its suitability for use with low-viscosity bioinks only and challenges in creating mechanically strong tissue constructs.^89^

### 5.2 Extrusion-Based Bioprinting

Extrusion bioprinting is the most used form of bioprinting due to its diverse nature, simplicity, and ability to work with a wide variety of biomaterials. Extrusion bioprinting is based on continuous extrusion of bioinks using pneumatic, piston-driven, or screw-driven systems to produce three-dimensional structures.^90^

The method supports bioinks with different viscosities such as hydrogels, cell-laden bioinks, growth factor-containing materials, and composites. This makes it possible to print large-scale tissue constructs, scaffold architectures with vascularization, and models of organs on clinically relevant scales. The main factors affecting cell viability are nozzle size, extrusion pressure, shear stress, bioink viscosity, extrusion speed, and cross-linking. Shear stress can harm cell membranes, but with an optimal nozzle shape and shear-thinning bioinks, cell viability can be enhanced postprinting.^22^

Extrusion bioprinting remains the most used technique because it accommodates high-viscosity bioinks and high cell densities. Pneumatic extrusion systems with thermal control improve bioink handling and printing accuracy. Extrusion printers offer multiple heads that facilitate printing of two different biomaterials concurrently, thus increasing complexity of tissue constructs. One of the strengths of extrusion bioprinting includes the production of high-density constructs and customized vasculature architecture. It has been used extensively in tissue engineering of bone, cartilage, neural tissue, cardiac tissue, and vascular tissue. On the other hand, extrusion bioprinting might exert significant shear stress to cells during passage through the nozzle, affecting cell viability. Optimization of parameters like pressure, nozzle diameter, extrusion speed, and rheology of material is therefore necessary. The extrusion method is still the most popular bioprinting technique due to its capability to use very viscous bioinks and high cell densities.^91^ Pneumatic extrusion methods that have thermally controlled components can maintain bioinks’ stability and increase deposition accuracy.^92^

### 5.3 Vat Photopolymerization-Based Bioprinting

Vat photopolymerization-based bioprinting, including stereolithography (SLA), digital light processing (DLP), and continuous liquid interface production (CLIP), has emerged as a high-resolution fabrication technique for tissue engineering. Compared with extrusion- and jetting-based approaches, it offers superior printing accuracy and surface quality while minimizing shear-induced cellular damage.^93^ The successful fabrication of constructs depends on appropriate selection of photo-cross-linkable bioinks, photoinitiators, and light exposure conditions. Because of its high spatial resolution, this technique has been widely applied in vascular, cartilage, neural, and organoid fabrication. The detailed principles and factors affecting cytocompatibility are discussed in Section 2.6.3.^94^

## 6 Biofabrication Strategies and Process Optimization

### 6.1 Multimaterial Bioprinting

Multimaterial bioprinting has proven to be one of the most significant inventions in modern-day biofabrication technology, as original tissue comprises cells with heterogeneous characteristics along with extracellular matrices and differential biochemical surroundings. Multimaterial systems help in deposition of more than one type of bioink in one structure to emulate the biological complexity found in native tissue laser-assisted bioprinting offers high resolution and nozzle-free deposition but has higher equipment costs.

Multimaterial systems make it possible to incorporate various cellular species along with biomaterials and bioactive compounds in certain regions within engineered tissues. In this way, complex tissues like the osteochondral interface, vascularized tissues, tumors, and even organ-on-a-chip technologies can be constructed using multimaterial systems. Engineering of multiple bioinks within a single construct enables the recreation of heterogeneous tissue structures and gradients also. Multimaterial systems improve biomimicry by distributing cells, growth factors, and biomaterials. Multimaterial extrusion, inkjet, and laser-assisted bioprinting technologies have brought about a revolution in bioprinting. The formation of strong interfaces between the different biomaterials is very crucial for stability.^88^

### 6.2 Coaxial and Gradient Printing

Coaxial bioprinting is a kind of extrusion-based bioprinting that employs concentric nozzles to create various biomaterial layers at one time. With such an advanced technique, it becomes possible to print hollow tubes, vascular grafts, and channels, which resemble blood vessels naturally.^95^ It played an important role in the development of vascular engineering because it was utilized to print concentric layers of cells and materials without losing their functionality and structure. Gradient bioprinting means printing gradients within a tissue or organ based on different biomaterial properties, such as composition, stiffness, number of cells, biochemical features, and so on. Such gradients are similar to those of natural gradients in tissue interfaces. This kind of bioprinting improves tissue growth, cell differentiation, and nutrition. Coaxial extrusion allows fabrication of hollow channels and vascular structures. Freeform coaxial bioprinting allows for the creation of hollow structures and vascular structures by coextruding core–shell bioinks simultaneously.^96^ Photo-cross-linking performed during the extrusion process increases structural stability and free-standing structure construction.

### 6.3 Vascularization Strategies

Despite many advancements, vascularization is still considered one of the biggest hurdles in tissue engineering, since thick tissues demand effective transportation of oxygen, nutrients, and waste products. The lack of proper vasculature causes areas in the center of engineered tissues to become hypoxic and necrotic.^97^ There are several methods that could be used to create vessels in engineered tissues such as sacrificial bioprinting, embedded printing, inclusion of endothelial cells, growth factor delivery, and coaxial bioprinting.^98^ One way to construct vascularized tissues is through the use of sacrificial bioinks, such as gelatin and Pluronic F127, where a network of hollow channels can be printed and subsequently extracted. In this way, researchers have managed to print thick vascularized tissues with the ability to preserve cell viability.^99^ The inclusion of endothelial cells, angiogenic factors, and a proper vascular architecture within engineered tissues contributes greatly to successful tissue maturation and implantation. Graphene oxide modifies bioink support endothelial cell growth and vascular graft fabrication. Endothelial progenitor cell-loaded scaffolds enhance angiogenesis and bone repair.^100^

### 6.4 Postprinting Maturation Techniques

After fabrication, there is a need for bioprinted tissues to mature in order to obtain viable tissue growth. This is where proliferation, differentiation, extracellular matrix deposition, and tissue organization occur. In relation to postprinting maturation, bioreactors provide nutrient delivery, oxygen supply, waste product removal, and mechanical stimulation. This results in more functional tissues with proper organizational structure. One of the postprinting maturation systems is the perfusion system, which is vital in the growth of vascularized tissues due to endothelial cell growth, stabilization of blood vessels, and tissue survival. Some recent developments also include the use of electrical, biochemical, and mechanical stimulation in order to enhance tissue development and function. The integration of vascularization techniques and postprinting maturation processes has increased the application of bioprinted tissues/organs in a clinical setting.^101^ Bioreactors provide mechanical stimulation that enhances tissue maturation and functionality. Cell culture conditions and bioreactor stimulation significantly influence tissue maturation and functional development after printing.

## 7 Next-Generation Bioinks and Recent Advancements in Bioink Development and Bioprinting Strategies

Along with the evolution of bioink technologies, there have been advances in bioprinting methodologies that are capable of producing biomimetic tissues. With advanced methods such as freeform reversible embedding of suspended hydrogels (FRESH), volumetric bioprinting, acoustic-assisted bioprinting, coaxial bioprinting, sacrificial bioprinting, and multimaterial bioprinting, it is now possible to produce complex tissue structures with enhanced accuracy, vasculature formation, and heterogeneous cellular organization that more closely resemble native tissues than those produced by conventional layer-by-layer bioprinting. These approaches enable the fabrication of complex tissues with hierarchical architectures, vascular networks, and heterogeneous cellular organization that more closely resemble native tissues than those produced by conventional layer-by-layer bioprinting. Consequently, the integration of advanced bioprinting strategies with next-generation bioinks represents an important direction toward clinically relevant tissue engineering and regenerative medicine applications.^102^

Advances in novel bioinks have made 3D bioprinting a simple printing technique. Bioinks not only serve structural purposes but are also functional in the regulation of cellular activities, tissue maturation, and biological signal transmission. In this context, novel bioink systems incorporate smart components, biomimetic material design techniques, and tissue-specific extracellular matrix components.^103–105^ In contrast to traditional hydrogels, the next generation of bioinks has been developed to concurrently fulfill the conditions of being printable, mechanically stable, biologically active, and able to undergo tissue remodeling for a lifetime. The main goal is to be able to engineer constructs that mimic native tissues both structurally and functionally and that support regeneration after being implanted.^106^ Some of the emerging technologies in the bioink are mentioned in Table 2.

**Table 2:** Emerging Technology of Bioink

**Emerging technology**	**Description**	**Major outcome**
**Organoid bioinks**	Bioinks containing organoids and stem cell derived mini organs	Enable patient-specific disease modeling and the regenerative medicine^112^
**Microgel based bioinks**	Bioinks consisting of the packed hydrogel microparticles	Shows excellent printability, shear thinning as well as cell protection^113^
**Living bioinks**	The bioinks containing genetically engineered living cells	Dynamic tissue engineering and the biosensing^114^
**Exosomes enriched bioinks**	The bioinks loaded with the extracellular vesicles and the exosomes	Enhanced angiogenesis and the tissue repair without direct cell transplantation^115^
**Immunomodulatory bioinks**	The bioinks responsible to create the host immune response	Reduction of inflammation and improving the implant integration^116^
**Organ on chip bioinks**	Bioinks for micro physiological systems and the organ on chip devices	The drug screening and the personalized medicine

### 7.1 Nanocomposite Bioinks

These nanocomposites contain nano-strengthening systems added to hydrogels that improve upon the poor mechanical behavior that is characteristic of biofabrication materials. The various types of nanomaterials that can be incorporated include carbon nanotubes, nanoclays, nanofibers, cellulose nanocrystals, graphene analogues, and other bioactive nanoparticles.^107–109^ Incorporating the nanomaterials results in improved compressive strength, elasticity, rheology, and shape fidelity without adversely impacting cellular compatibility.This allows us to create mechanically robust structures that can be used in load-bearing applications such as bone and osteochondral tissue engineering. Carbon nanotube nanocomposites within hydrogels show increased strength of matrix-like scaffolds while retaining their porosity and capacity to grow cells. These nanocomposite structures increase the electrical conductivity and may also help to promote cell differentiation.^110^ Another advantage provided by nanocomposites is an extended biofabrication window due to a combination of high print fidelity and good biological behavior. This is possible because of the shear thinning nature of these bioinks. Nanoparticles act not only as reinforcement filler materials but also as multifunctional particles for bioactivity, electrical conductivity, and sustained release functions.^111^ Nanocomposite bioinks offer better viscosity, shear thinning, and mechanical performance at once. Nanoparticles improve the rheological behavior, mechanical strength, conductivity, and regenerative potential. Graphene oxide acts as a multifunctional nanoelement rather than as a simple reinforcing filler.

### 7.2 Decellularized ECM Bioinks

One of the most biologically important approaches to tissue engineering is the use of decellularized extracellular matrix bioinks. They are made by extraction of cellular material with retention of extracellular matrix proteins, growth factors, glycosaminoglycans, and biochemical messengers.^117,118^

One of the main benefits of using dECM bioinks is the opportunity to generate tissue-specific environments that simulate native biological niches. The structure of the extracellular matrix in these materials provides the necessary biochemical and biomechanical signals that regulate the behavior of cells. Tissue-specific decellularized bioinks have already been produced from cardiac tissue, cartilage, liver, skin, skeletal muscle, pancreas, blood vessels, cornea, kidney, adipose tissue, and neural tissues. Such materials can induce characteristic reactions in cells that cannot be obtained by other methods. Current studies are concentrated on increasing the printability and mechanical properties of dECM formulations by employing reinforcement techniques and combinations with other biomaterials. These materials allow achieving increased structural integrity without loss of biological advantages of the native extracellular matrix.^88^ Moreover, dECM bioinks are able to produce the specific biological signals that can stimulate the generation of organ-specific tissues and remodeling of tissue structures after their implantation.^119^ ECM bioinks replicate original tissue microenvironments more accurately than conventional hydrogels. dECM bioinks provide tissue-specific microenvironments that enhance cell behavior and tissue-specific differentiation. Tendon-derived dECM bioinks demonstrate excellent potential for musculoskeletal tissue engineering applications.

### 7.3 Stimuli-Responsive and Smart Bioinks

Stimuli-responsive bioinks are usually created based on the dynamic response of such systems to external stimuli due to their physical and biochemical modifications. The materials offer an additional degree of adaptability that better mimics natural biological tissues.^120^ Temperature-responsive systems involve a sol–gel transition for printing and scaffold stability under physiological conditions. Such a mechanism provides a positive impact on handling properties and integrity of structures after fabrication; pH-responsive materials allow controlling their degradation rate, swelling, and release of active molecules based on physiological conditions in the specific area. This feature makes pH-responsive systems useful for drug-delivery methods. Photoresponsive silk fibroin bioinks enable controlled gelation and shape stabilization. Light-responsive bioinks involve light-induced changes that control cross-linking, architecture, and properties of printed materials. Light-responsive systems are commonly used in biofabrication and 4D printing processes.

### 7.4 4D Bioprinting Bioinks

The beginning of 4D bioprinting involves adding another dimension to the process of biofabrication. In contrast to static 3D printed models, 4D bioinks permit the creation of evolving structures through programmed reactions to certain stimuli after biofabrication.^121^ Bioinks used in 4D biofabrication often consist of smart materials with the capacity to react to heat, moisture, acidity, magnetic fields, light, and electricity, among other environmental conditions. Reactions trigger changes in the shape, structure, or biological activity as a function of time. Shape-morphing hydrogels and biomimicking swelling systems have been known to self-fold, self-assemble, and transform into structures that mimic developmental processes seen in live tissues.^122^ 4D bioprinting introduces dynamic structures capable of changing shape or function over time after implantation. Transformation over time permits the engineered tissue to respond better to changing physiological conditions, leading to increased tissue maturity and better regenerative functions.

### 7.5 AI-Designed Bioinks

The use of artificial intelligence is one of the ways of developing and optimizing bioinks much faster than other methods. The process of bioink formulation involves a lot of empirical work that consumes much time, effort, and resources. However, artificial intelligence offers data-based solutions for the problem at hand.^123^ Machine learning algorithms can study data sets with data on various aspects of bioinks, such as rheology, printability, viscosity, cross-linking ability, degradability, mechanical performance, and biological effects. Using artificial intelligence in developing bioinks also means that scientists will be able to optimize the printing process by changing parameters like extrusion pressure, nozzle size, printing speed, and the structure of the printed scaffolds. Another advantage of AI-supported bioink production is that predictive modeling makes it possible to predict printing results using simulations prior to performing actual experiments, which saves materials and money. Recently, a few research papers have highlighted that biofabrication using the concept of artificial intelligence has been applied to make personalized tissue constructs. Artificial intelligence can optimize the print settings, predict the printability, and avoid experiments through trial and error.^59^ The application of AI models in choosing suitable bioinks is possible through this process.

#### 7.5.1 Self-Healing Bioinks

Self-healing bioinks comprise a highly sophisticated type of dynamic biomaterials, which can autonomously recover from mechanical deformations and injuries. The mechanism of self-healing is based on the use of reversible dynamic covalent bonding (such as Schiff base and boronate ester bonds) and noncovalent interactions (such as hydrogen bonding, host–guest interactions, ionic bonding, and supramolecular assembly). The self-recovering property allows them to deform multiple times during the printing process, while maintaining the printability and viability of cells. Moreover, not only improved shape recovery and enhanced mechanical stability but also a dynamic environment created by self-healing bioinks can stimulate cell migration, proliferation, extracellular matrix production, and tissue regeneration. The self-healing property makes these bioinks highly applicable for engineering cartilaginous, skin, cardiovascular, neural, and musculoskeletal tissues, which are prone to mechanical stress. Current developments in this field involve the integration of self-healing behavior with stimuli-responsive and conductive material to create highly functional bioinks.^102^ Examples: GelMA, oxidized alginate-gelatin hydrogels, hyaluronic acid–based hydrogels, supramolecular peptide hydrogels.

#### 7.5.2 Cell Aggregate Bioinks

In cell aggregate bioinks, which can also be described as scaffold-free bioinks, the basic components used are multicellular spheroids, organoids, tissue strands, or cell aggregates instead of typical hydrogel scaffolds. The cell aggregate bioinks capitalize on the natural tendency of cells to form aggregates and produce an extracellular matrix, thus imitating the natural tissue structure and cell–cell interactions closely. Due to the absence of or very little exogenous biomaterials, scaffold-free constructs do not pose any worries relating to biomaterial degradation, cytotoxicity, or inflammation but rather help in maturing and improving the physiological functions of the tissues. Cell aggregate bioinks have exhibited tremendous potential in the production of tissues that are highly cellular, such as liver tissues, cardiac muscle, cartilage, and vascular tissues. Nevertheless, some problems, such as poor mechanical strength, difficulty in handling, inadequate nutrient diffusion, and vascularization, have limited the use of cell aggregate bioinks clinically. At the moment, efforts are being made to incorporate cell aggregates into supporting biomaterials and maturation and vascularization systems in bioreactors.^1^ Examples: mesenchymal stem cell spheroids, cardiac spheroids, hepatic organoids, and tissue strands.

#### 7.5.3 Gene-Activated Bioinks

Gene-activated bioinks are a new generation of biofunctional materials developed to provide therapeutic delivery of nucleic acids such as plasmid DNA, mRNA (mRNA), small interfering RNA (siRNA), microRNA (miRNA), and CRISPR/Cas-based gene editing systems inside bioprinted constructs. Unlike the provision of exogenous proteins or growth factors, gene-activated bioinks allow the targeted expression of therapeutic genes from encapsulated or host cells, providing prolonged biological activity and improved tissue regeneration. Gene-activated bioinks are capable of regulating cell proliferation, differentiation, angiogenesis, osteogenesis, and immunomodulation through the controlled expression of therapeutic genes, thus becoming especially useful in bone, cartilage, neural, and vascular tissue engineering. To enhance the efficiency of gene delivery and protect nucleic acids from degradation, they are usually delivered into bioinks using nanoparticles, liposomes, or polymeric materials prior to bioprinting. While offering significant therapeutic possibilities, there are still many challenges associated with low transfection efficiency, controlled release kinetics, biosafety issues, and other regulation-related concerns that limit the clinical application of gene-activated bioinks. Integrating gene editing systems with smart bioinks is expected to provide advanced personalized and precise therapeutics.^1^ Examples: VEGF-plasmid-loaded bioinks, BMP-2 gene-activated hydrogels, siRNA-loaded GelMA bioinks, CRISPR/Cas9-modified hydrogels.

## 8 Applications of Bioinks

Bioinks have become important ingredients for modern 3D bioprinting because they allow the production of tissue-like structures with live cells, extracellular matrix materials, growth factors, and other bioactive substances (Figure 2). Development of bioink technology has opened new opportunities for using these ingredients beyond regenerative medicine to areas such as disease modeling, personalized medicine, and drug screening. The biomimicking capabilities of bioinks have greatly enhanced tissue functions, cell alignment, and therapeutic effects.

**Figure 2:**
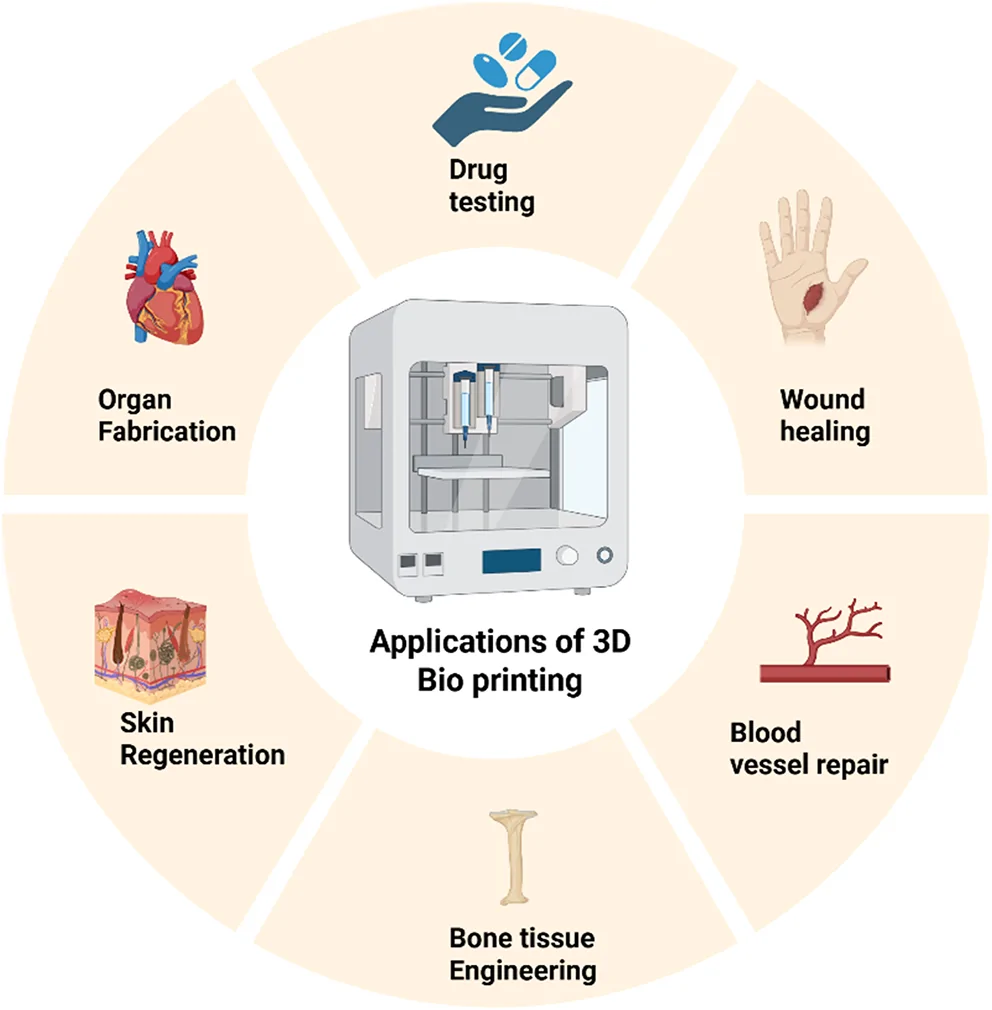
Applications of 3D Bioprinting (Figure created using BioRender).

### 8.1 Skin Bioprinting

Skin bioprinting is one of the most clinically promising uses of bioinks since there is an increasing number of patients with burns, chronic skin lesions, diabetic ulcers, and traumatic skin damage. Bioinks with keratinocytes, fibroblasts, collagen, gelatin, hyaluronic acid, and other extracellular matrix proteins have been used successfully to produce skin grafts that mimic the natural architecture of the skin. ECM-derived bioinks can recreate skin-like microenvironments and improve wound healing potential. Cell adhesion, proliferation, and deposition of the extracellular matrix on collagen-based bioinks favor cell activity; thus, they are good materials for the regeneration of epidermis and dermis. In addition, the inclusion of growth factors and stem cells makes these bioinks excellent in wound healing promotion and angiogenesis. In recent times, researchers have employed skin-derived decellularized extracellular matrix bioinks to recapitulate tissue-specific biochemical cues that promote maturation and regeneration of tissues. Such bioinks display increased tissue integration and faster wound closure. Skin bioprinting technology has become more advanced to produce tissue grafts customized for patients.^120^

### 8.2 Bone and Cartilage Tissue Engineering

Bone and cartilage regeneration can be considered one of the most important where there is a significant need for bioinks since standard approaches do not allow for the restoration of the whole integrity of tissues. The bioinks to be used for musculoskeletal applications should provide adequate mechanical properties and induce osteogenic and chondrogenic differentiation. Among promising candidates for bone tissue regeneration, there are hydrogel-based bioinks with additives such as hydroxyapatite, β-tricalcium phosphate, bioactive glass, nanoclay, and collagen. In addition, composite bioinks enhanced by carbon nanotubes, graphene derivatives, and cellulose nanomaterials show promise in providing high mechanical stability in the long run as well as enhancing bone regeneration. Hydrogels for cartilage regeneration should provide biological and mechanical cues needed for inducing chondrogenesis. Hyaluronic acid, gelatin methacrylate, collagen, and cartilage-derived dECM are among promising additives. They create an environment mimicking native cartilage tissue. Akermanite scaffolds demonstrate superior osteogenic activity and higher compressive strength than β-TCP scaffolds.^124^ Zn^2+^-enhanced SFMA/HisMA/nHAP scaffolds promote vascularized bone regeneration. Hydroxyapatite/chitosan/collagen constructs show strong potential for dental bone regeneration. Recently, it has been shown that dECM-based cartilage bioinks exhibit higher cell responses as compared with conventional materials because of preserving native ECM structures, advances in bioprinting technology have made it possible to create constructs that exhibit enhanced osteogenesis and chondrogenesis capabilities. Photopolymerized hydrogels show significant promise in terms of load-bearing tissue-engineering applications. Bioactive periodontal scaffolds can regenerate the periodontal ligament and alveolar bone.^39^

### 8.3 Neural Tissue Engineering

The design of bioinks for the construction of neural tissues is one of the most difficult tasks due to the complexity of neural tissue architecture and poor regeneration potential. Neural bioinks should ensure neuronal viability, axon extension, and electrical communication between neurons. Bioprinted scaffolds can remodel the neuromodulatory microenvironment and improve the bone regeneration through nerve–bone interactions.^125^

Hydrogels composed of neural stem cells, extracellular matrix proteins, neurotrophins, and conductive nanomaterials have proven to be useful for neuronal regeneration. The use of these bioinks promotes neuronal differentiation and development of neuronal network connections. Bioactive materials are capable of regulating neural pathways, contributing to improved tissue regeneration.^48^ Hydrogels loaded with graphene and carbon nanotubes have been reported as beneficial because of increased electrical properties and suitable conditions for cell survival. In particular, these bioinks promote neuronal attachment and synaptic interactions. In addition, brain-derived decellularized extracellular matrices serve as bioinks, which mimic natural biochemical cues for neuronal development.

### 8.4 Cardiac Tissue Engineering

However, heart-related diseases continue to be among the leading causes of death, and there is an increasing need for artificial tissues and regenerative therapies. Myocardium bioinks seek to mimic the properties of the natural myocardium structure, composition, and electrophysiology. Myocardium bioinks made up of cardiomyocytes, endothelial cells, collagen, fibrin, gelatin methacrylate, and cardiac decellularized extracellular matrix create physiological conditions supporting cell survival and functionality. Decellularized extracellular matrix bioinks retain tissue-specific components promoting cardiomyocytes’ maturation and electrical coupling, which results in better performance than typical hydrogels. Photocross-linked gelatin/PEGDA hydrogels support heart valve cell encapsulation with high viability. Furthermore, adding conducting materials into bioinks together with vascularization methods increases conductivity and delivery of nutrients throughout the tissue. Bioprinting of cardiac tissues has been proven to demonstrate great potential in repairing damaged cardiac muscles, modeling diseases, screening drugs, and regenerative medicine.^126^

### 8.5 Tumor Models

The construction of three-dimensional cancer models is another key application of bioink materials that has revolutionized research in oncology. Two-dimensional cancer models cannot reproduce cellular interactions and biochemical gradients characteristic of real tumors. Advanced hydrogel bioinks enable the creation of physiologically relevant tumor microenvironments for cancer research.^127^ Dipeptide hydrogel scaffolds provide a promising platform for constructing complex 3D tissue models and disease models.^128^ Cancer bioinks that incorporate tumor cells into matrices consisting of collagen, gelatin, hyaluronic acid, and decellularized extracellular matrix hydrogels simulate specific tumor environments. 3D tumor models offer scientists an opportunity to study the biology of tumors under physiological conditions and increase the validity of drug testing. Advances in multimaterial bioprinting techniques have made it possible to model tumors incorporating the spatial distribution of cancer, stromal, immune, and vascular compartments. Individualized tumor models based on patient-specific bioink materials facilitate personalized medicine and tailored therapy options.^102^

### 8.6 Drug Screening and Pharmaceutical Applications

One of the most rapidly developing uses of bioinks is drug screening, which occurs due to the deficiencies inherent in traditional animal models and cell cultures on flat substrates. Conventional systems have often failed to forecast human reactions, resulting in drug failure during development processes. Tissue constructs are produced through bioprinting based on advanced bioinks represent highly realistic models because they can mimic natural tissue organization, matrix, and cell-to-cell contact. As a result, the quality of drug effectiveness and toxicity estimation has increased greatly. Bioprinted tissues provide physiologically relevant environments for evaluating drug efficacy and toxicity.^129^ Models of the livers, hearts, neural tissues, skin, and tumors created via bioinks have already shown great potential in pharmacology and disease modeling. They not only provide more accurate predictions but also decrease the use of animals in experiments. The combination of artificial intelligence and biofabrication in drug screening allows for automatic optimization of models and analysis of drug responses. Bioprinted tissue models provide more predictive platforms for evaluating drug efficacy and toxicity compared with conventional 2D cultures. With recent developments in personalized bioprinting, it is now possible to add patient-specific cells to bioinks, enabling personalized drug testing and therapies. In summary, there are more emerging applications for bioinks in regenerative medicine, tissue engineering, biomedical studies, and pharmaceutical research. Improvements in dECM bioinks, bioinks with nanocomposites, smart biomaterials, and AI-assisted bioprinting can be anticipated in the future [Ref].

## 9 Translational Considerations in Bioink Development

Successful translation of bioinks into clinical use involves more than merely excellent printability and biological properties. For bioinks to transition from lab work to industrial and clinical applications, they should comply with certain regulatory requirements, scalable manufacture, quality assurance procedures, storage conditions, and economic feasibility considerations. While significant achievements have been reached regarding the development of biomaterials and printing systems, there is still much room for improvement before bioinks could become standard tools in medicine.^130,131^

### 9.1 Regulatory Landscape

Regulatory approval continues to be one of the key barriers in the translation of bioinks due to their combination of properties from biomaterials, medical devices, biologics, and cellular therapy products. The regulatory pathway is dependent on the jurisdiction. Bioink constructs should be evaluated in terms of their safety, effectiveness, reproducibility, sterility, biocompatibility, and long-term efficacy before being introduced into the clinics. Extensive preclinical and clinical validation is necessary to demonstrate compliance with the requirements set by the regulatory agencies. Standardization and regulatory approval remain major challenges before widespread clinical adoption of bioprinted tissues. The absence of consensus international regulations applicable to bioprinted tissues and organs is another key issue in the process of developing standards for this emerging field. Regulations have been elaborated based on the principles governing conventional medical devices and tissue-engineered products and might not cover all aspects of bioprinted products. Current discussions highlight the importance of establishing an international regulatory framework for accommodating the rapid development in biofabrication. Regulatory frameworks remain underdeveloped for many bioprinted products, particularly cell-laden constructs.^132^ The development of artificial intelligence and autonomous manufacturing may require special regulatory guidelines to validate algorithms and decision-making processes used in these novel systems.

### 9.2 GMP and Manufacturing Requirements

Compliance with Good Manufacturing Practice (GMP) guidelines is vital to large-scale production of bioink-based constructs. GMP systems provide assurance that the manufacture of goods produces products with predetermined quality attributes. The manufacture of bioinks calls for strict quality control during raw materials selection, sourcing of cells, biomaterial processing, sterilization process, environmental control, and quality assurance protocols, as variability in any of these factors can affect the final product performance.^133^ Modern biofabrication platforms have emphasized the need to incorporate automated biofabrication robots, biosensors, artificial intelligence, and closed-loop monitoring systems in the production process to avoid mistakes and ensure product consistency. Self-driving bioprinting laboratories have been suggested as an option to integrate automation, biosensing, closed-loop monitoring, and AI systems, as well as enable autonomous manufacture of bioinks, construct manufacturing, maturation of tissues, and evaluation. Also, the facility will be required to provide a sterile manufacturing environment for the production process, as well as standardized workflow processes.^134^ GMP compliance in manufacturing will be key to accelerating regulatory approvals of the products. Translational bioinks require scalable manufacturing processes, reproducibility, sterility, and regulatory compliance. Sterility, quality control, and reproducibility are critical for clinical-grade bioink production.

### 9.3 Scalability and Standardization

Scalability continues to represent one of the biggest challenges for the successful implementation of bioprinted tissues and organs in the clinic. While there have been many examples of bioinks that show great promise in the lab setting, there continue to be limitations when scaling up production to industrial levels. Scaling up will involve standardizing processes for synthesizing biomaterials, culturing cells, formulating bioinks, cross-linking, bioprinting, and postprocessing. Variability in material characteristics and biological composition may affect the ability to produce a product in a repeatable fashion. This becomes especially important since bioinks frequently contain living cells, as well as extracellular matrix proteins, growth factors, and other biological molecules. Standardizing quality control measures will thus become necessary.^56^

The development of advanced biofabrication techniques, digital manufacturing capabilities, and AI-optimized production will likely facilitate this process by allowing for ongoing process monitoring and adaptive control.

In addition, the use of multimaterial bioprinting and personalized approaches to tissue fabrication will have to be implemented within an industrialized manufacturing process.^135^ Commercialization requires the standardization of bioink formulation, printing protocols, and quality control procedures. Large-scale production of bioinks requires standardization of formulations and reproducible manufacturing protocols.^48^

### 9.4 Storage and Stability

Next, there are key practical concerns for commercialization of bioink, such as storage and long-term stability. Common hydrogel bioinks have short shelf life, as they degrade, become microbiologically contaminated, lose their rheological properties, and lose biological functional properties during storage.^136^ Recently, researchers have been working on improving the preservation properties of bioinks. The bioinks formulated with glycerol hydrogel have exhibited outstanding shape retention properties, intrinsic antibacterial properties, and regulation of water state in the bioink to improve its resistance to microbial contamination over a long-term period. One of the key advances has been the creation of bioinks that are intrinsically cryopreservable and can preserve the viability of cells during ultralow-temperature storage. This type of system can inhibit the formation of ice crystals while transporting and in long-term storage of bioprinted tissues. The cryopreservation technologies are anticipated to become a critical component of future clinical applications as a means to create a centralized manufacturing and distribution system for bioink-based products to healthcare facilities around the world. Thus, further advancement of bioink stability, storage, and logistics is still crucial for commercialization and large-scale application. Storage conditions influence parameters such as viscosity, printability, and biological activity of bioinks.^137^

### 9.5 Cost-Effectiveness and Commercialization Potential

One of the important factors for successful commercialization is economic feasibility. Although great progress has been made in the technology, some advanced bioinks are still costly as they require specialized equipment, high production costs, expensive biomaterials, and labor-intensive manufacturing processes.

Expenses for production can significantly rise with the use of patient-specific cells, decellularized extracellular matrices, growth factors, and customized biomaterial formulations. In addition, there are the costs of maintaining facilities that are GMP-compliant as well as the costs involved in conducting extensive quality control testing. But the rise and improvements of automation, AI, robotics, and high-throughput biofabrication are predicted to drive down manufacturing costs and increase manufacturing efficiency. Autonomous bioprinting systems can greatly reduce the need for labor and enhance the repeatability and efficiency of printing. Bioinks can also be used in commercial applications other than regenerative medicine, such as those in pharmaceutical testing, disease modeling, personalized medicine, cosmetic testing, and organ-on-chip technologies. While full-functioning bioprinted organs are still under development, these applications offer near-term applications for commercialization. In addition, personalized tissue fabrication approaches were also shown to have great market potential due to their ability to deliver patient-specific therapies that yield improved therapeutic outcomes and minimal immune rejection risks.^138,139^ In conclusion, the integration of regulatory compliance, GMP manufacturing, scalable production, long-term storage, and economically viable commercialization strategies will be key to the translation of bioinks from lab to clinic. As automation, artificial intelligence, standardized manufacturing, and smart biomaterials continue to develop, the clinical use of bioink-based technologies should continue to accelerate and will become a key cornerstone of future regenerative medicine. Simple, physically cross-linked bioinks reduce manufacturing complexity and production cost.

## 10 Future Perspective

3D bioprinting enables scientists to produce highly complex tissues through careful designing of scaffolds, alignment of cells, and deposition of biomaterials. In addition to that, the rapid advancement in bioink, nanomaterial, and growth factor technologies, the possibility of creating vascularized tissues, personalized medicine, demand for artificial organs, etc., are among key factors that have made this technology one of the major trends in contemporary biomedical research. Moreover, today this technology is widely used outside the sphere of healthcare, including the pharmaceutical industry, drug testing, artificial food manufacturing, and more. Development of innovative concepts like smart bioinks and 4D printing will hold much promise in the future. 3D bioprinting is thus an important technology for the future of regenerative medicine because conventional tissue engineering methods have been unable to effectively simulate the complex hierarchical structures, heterogeneity of cell populations, vascularization, and structure of the extracellular matrix in natural tissues. Pore engineering increases the effectiveness of oxygen diffusion, delivery of nutrients, elimination of waste products, and the viability of cells in engineered tissues. Moreover, advanced bioprinters play an important role in the manufacture of personalized medical devices. Conductive nanomaterials, growth factors, platelet-rich plasma, and metabolically active biomaterials enhance the regeneration of tissues and enable cell-to-cell communication. In addition, through 3D bioprinting, functional tissue models for research in diseases, pharmacology, personalized medicine, and regenerative therapy may be created; thus, minimizing dependence on donated tissue samples and animals in experiments. Overall, with bioinks, smart biomaterials, and printing technology, 3D bioprinting has the potential to revolutionize biomedical and pharmaceutical science.

## 11 Conclusion

In tissue engineering and regenerative medicine, 3D bioprinting is a revolutionary technology that has become a focus of research, and a major component of 3D bioprinting is the development of bioinks, which are materials used to print bioconstructs. Bioinks have now evolved from basic hydrogel-based formulations to more sophisticated, next-generation bioinks that contain a variety of stimuli-responsive materials, growth factors, decellularized extracellular matrix components, stem cells, and nanomaterials. These innovations have resulted in greatly enhanced printability, durability, biocompatibility, and tissue-appropriate functionality. Even though significant progress has been made, there are still significant hurdles including, but not limited to, vascularization, long-term stability, scalability, regulatory approval, and clinical translation. New techniques such as AI-guided bioink design, smart bioinks, and 4D bioprinting hold promise for addressing current challenges and facilitating the fabrication of more complex, dynamic, and patient-specific tissues. To achieve the full clinical promise of biofabricated tissues and organs, ongoing interdisciplinary research, bridging materials science, biology, engineering, and computational technologies, will be critical.
